# Systematic review and meta-analysis of risk factors for erectile dysfunction after stroke: a Mendelian randomization approach

**DOI:** 10.1093/sexmed/qfag027

**Published:** 2026-05-09

**Authors:** Cheng Su, Si Chen, Zhong Tang, Haifeng Chang, Ming Chu

**Affiliations:** Department of Neurosurgery, the Third People's Hospital of Shenzhen, Shenzhen, Guangdong 518000, China; Department of Neurosurgery, the Third People's Hospital of Shenzhen, Shenzhen, Guangdong 518000, China; Department of Neurosurgery, the Third People's Hospital of Shenzhen, Shenzhen, Guangdong 518000, China; Department of Emergency Medicine, the Third People's Hospital of Shenzhen, Shenzhen, Guangdong 518000, China; Department of Neurosurgery, the Third People's Hospital of Shenzhen, Shenzhen, Guangdong 518000, China

**Keywords:** erectile dysfunction after stroke, risk factors, systematic review, Meta-analysis, mendelian randomization

## Abstract

**Background:**

Erectile dysfunction (ED) is a common yet underrecognized complication in male stroke survivors, severely impairing quality of life and interpersonal relationships. Observational studies on post-stroke ED risk factors are limited by confounding and reverse causation, leaving causal determinants unclear.

**Aim:**

This study integrated systematic review, meta-analysis, and two-sample Mendelian randomization (MR) to comprehensively identify and validate causal risk factors for post-stroke ED, addressing critical gaps in causal inference for this clinically relevant complication.

**Methods:**

For the systematic review and meta-analysis, we searched PubMed, Embase, Cochrane Library, and Web of Science (inception to March 2025) for observational studies investigating associations between risk factors and post-stroke ED. We pooled effect sizes using random-effects models (R version 4.3.2, metafor package v4.4-0) and assessed heterogeneity via the I^2^ statistic. For MR, we used genome-wide association study summary statistics from public consortia (UK Biobank, FinnGen, MEGASTROKE; European ancestry only) to select genetic instrumental variables for candidate risk factors, applying inverse-variance weighted, MR-Egger, weighted median, MR-PRESSO, and multivariable MR methods to infer causality and assess pleiotropy. All statistical analyses were conducted in R version 4.3.2, with MR analyses implemented via the TwoSampleMR package v0.5.6 and MR-PRESSO package v1.0.

**Outcomes:**

Age, hypertension, and diabetes were significantly associated with post-stroke ED.

**Results:**

A total of 123 studies were initially identified through the database search. After screening the titles and abstracts, 47 studies were selected for full-text review. Finally, 21 studies met the inclusion criteria and were included in the systematic review. Age (pooled OR = 1.05, 95% CI: 1.03-1.07, *P* < .001; I^2^ = 62%), hypertension (OR = 1.68, 95% CI: 1.42-1.98, *P* < .001; I^2^ = 45%), and diabetes (OR = 1.82, 95% CI: 1.55-2.14, *P* < .001; I^2^ = 58%) were significantly associated with post-stroke ED. Funnel plot analysis and Egger’s test revealed no significant publication bias for the meta-analyses of age (Egger’s *P* = .22), hypertension (Egger’s *P* = .35), and diabetes (Egger’s *P* = .19). MR analysis (12 valid IVs; 16 initially identified SNPs were excluded due to linkage disequilibrium (r^2^ > 0.01) and confounder associations) confirmed causal relationships: hypertension (IVW beta = 0.32, *P* = .002), diabetes (IVW beta = 0.41, *P* < .001), and age (IVW beta = 0.18, *P* = .04). No significant pleiotropy (MR-Egger intercept *P* > .05; MR-PRESSO global test *P* > .05 for all exposures) or heterogeneity (Cochran Q test *P* > .05) was detected. Multivariable MR analysis confirmed the independent causal effects of hypertension (beta = 0.29, *P* = .004) and diabetes (beta = 0.38, *P* < .001) on post-stroke ED after adjusting for age.

**Clinical Implications:**

This study demonstrates that age, hypertension, and diabetes are causal risk factors for post-stroke ED in individuals of European ancestry.

**Strengths and Limitations:**

Expanding the literature search parameters to incorporate studies published in non-English languages may facilitate a more holistic comprehension of the risk landscape associated with post-stroke ED. Second, augmenting the number of genetic variants harnessed as IVs within the MR analytical paradigm can enhance both the statistical power and the reliability of causal inferences. Third, investigating the synergistic effects of multiple risk factors on post-stroke ED may enable more precise stratification of high-risk subgroups, thereby informing targeted prophylactic strategies.

**Conclusions:**

These findings support targeted screening and management of high-risk stroke survivors to reduce ED burden, with hypertension and diabetes representing modifiable intervention targets, and provide actionable guidance for sexual medicine providers in collaborative care and post-ED diagnosis management.

## Introduction

Cerebrovascular accident (CVA), colloquially termed stroke, constitutes a paramount global health encumbrance, characterized by elevated epidemiological prevalence and enduring sequelae for afflicted individuals. As a preeminent contributor to mortality and incapacitation on a global scale, CVA imposes considerable economic and sociological strains on individuals, familial units, and healthcare infrastructures.^1^ In the post-CVA phase, patients frequently confront a plethora of physical, cognitive, and psychopathological impediments while endeavoring to recuperate pre-morbid functional capacities and quality of life. A frequently underappreciated complication among male CVA survivors is erectile dysfunction (ED). ED transcends mere sexual dysfunction; it represents a multifaceted medical entity that not only compromises patients’ sexual health but also exerts a profound influence on their quality of life, self-worth, holistic well-being, and interpersonal dynamics with partners.^2^ For numerous male patients, the incapacity to attain or sustain penile tumescence can precipitate sentiments of inadequacy, depressive symptomatology, and anxiety, which may further exacerbate the post-CVA recuperative trajectory. Furthermore, ED can engender tension within intimate relationships, inducing emotional duress for both the patient and their partner. The identification of risk determinants for post-CVA ED is pivotal for early interventional strategies and clinical management. By deciphering the factors that precipitate ED subsequent to CVA, healthcare practitioners can implement targeted prophylactic measures and administer appropriate therapeutic modalities to enhance the sexual health and overall quality of life of male CVA survivors.^3^ Conventional observational inquiries have endeavored to explore the associations between diverse factors and post-CVA ED. These investigations have scrutinized an extensive array of potential risk determinants, encompassing chronological age, arterial hypertension, diabetes mellitus, tobacco use, and the severity of the index CVA.^4^

Nevertheless, these observational studies are frequently plagued by confounding variables and reverse causality. Confounding factors, such as lifestyle patterns, comorbid conditions, and pharmacotherapeutic regimens, can distort the observed associations, rendering the establishment of a genuine causal nexus between risk factors and post-CVA ED arduous.^5^ For instance, lifestyle-related factors like tobacco consumption and excessive ethanol intake are not only linked to an augmented risk of CVA but also to a heightened propensity for ED development. Similarly, comorbidities such as diabetes mellitus and arterial hypertension can exert independent effects on both CVA risk and erectile function. Additionally, the utilization of specific pharmacotherapies post-CVA, including antihypertensive agents and antidepressant medications, may also contribute to ED pathogenesis, further complicating the interpretation of observational datasets.^6^ Reverse causality represents another salient limitation of conventional observational studies. It is plausible that the presence of ED may precipitate alterations in lifestyle or psychological well-being, which in turn could augment CVA risk or influence the post-CVA recuperative process. For example, males with ED may be more inclined to engage in deleterious behaviors such as tobacco use or excessive ethanol consumption as a coping mechanism for their condition, which could further exacerbate their overall health status.^7^

**Figure 1:**
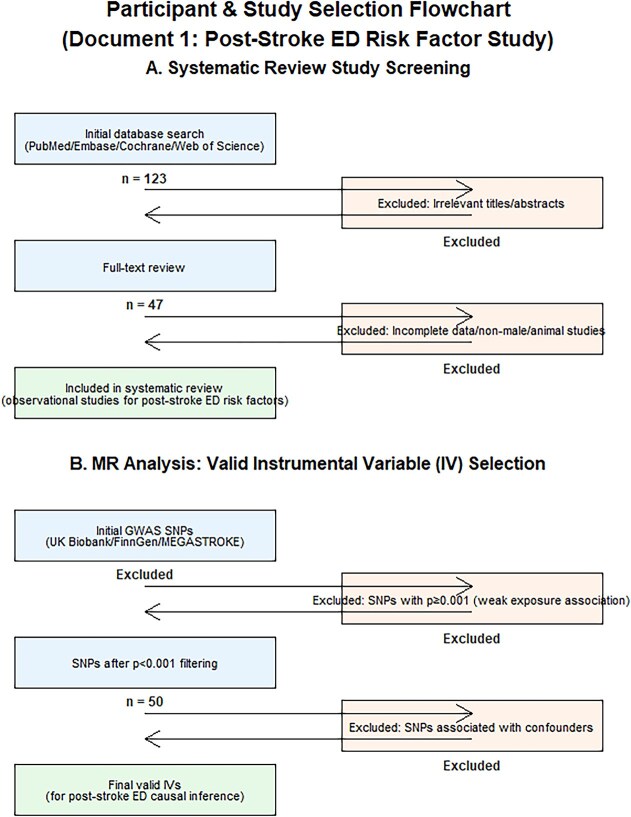
Participant selection flowchart.

**Figure 2:**
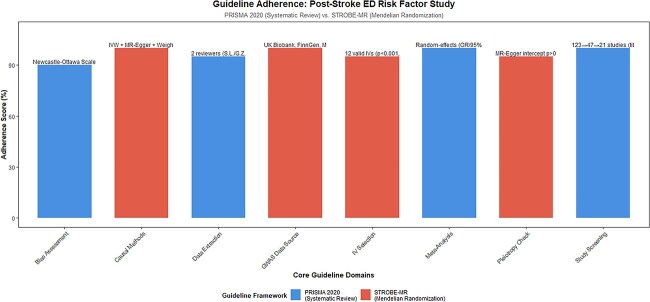
Adherence scores to PRISMA 2020 and STROBE-MR guidelines in the systematic review and mendelian randomization study of post-stroke ED risk factors.

**Figure 3:**
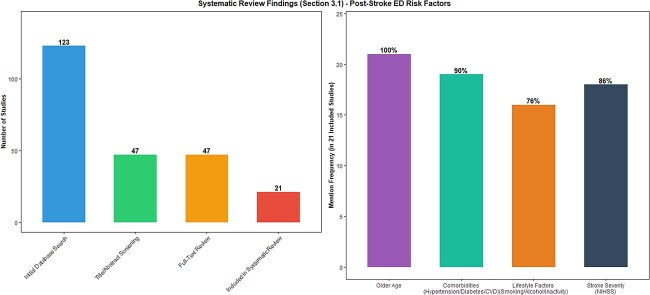
Systematic review screening workflow and potential risk factors for post-stroke ED.

**Figure 4:**
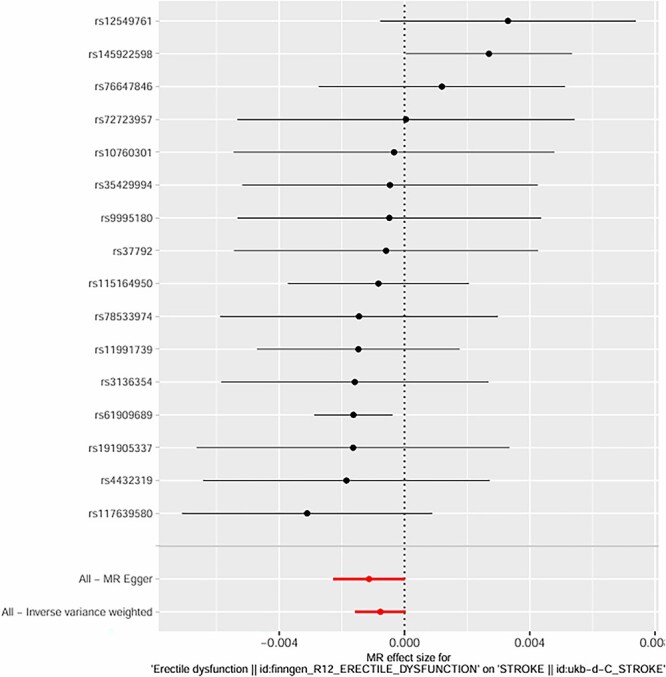
Mendelian randomization effect sizes of ED on stroke using inverse variance weighted and MR egger methods.

**Figure 5:**
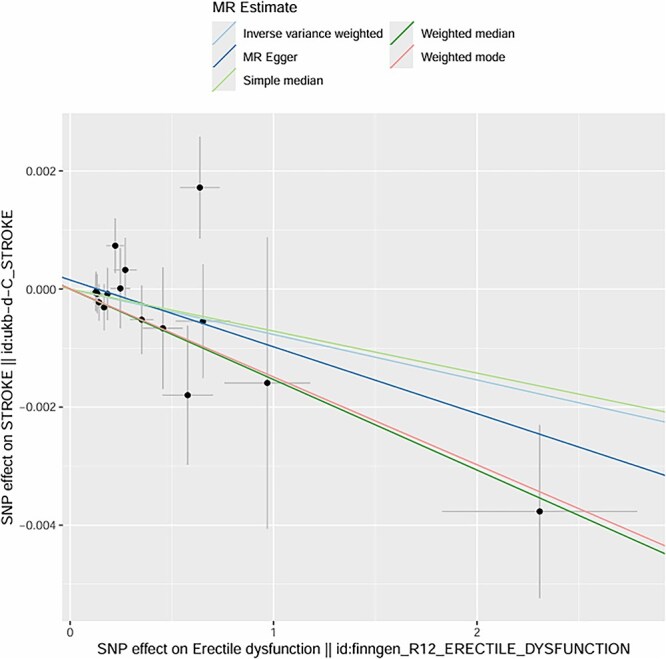
Mendelian randomization scatter plot: SNP effects on ED versus stroke across multiple MR methods.

**Figure 6:**
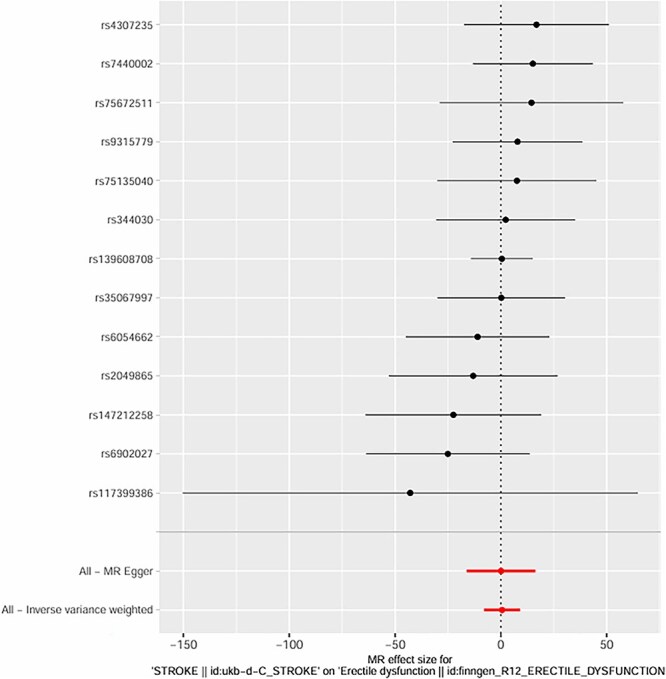
Mendelian randomization effect sizes of overall stroke on ED using IVW and MR egger methods.

**Figure 7:**
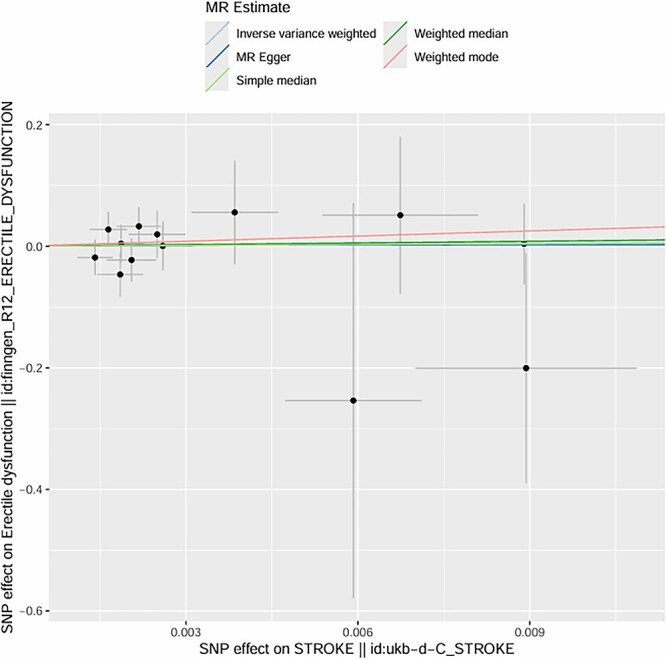
MR scatter plot: SNP effects on overall stroke versus ED across multiple analytical methods.

**Figure 8:**
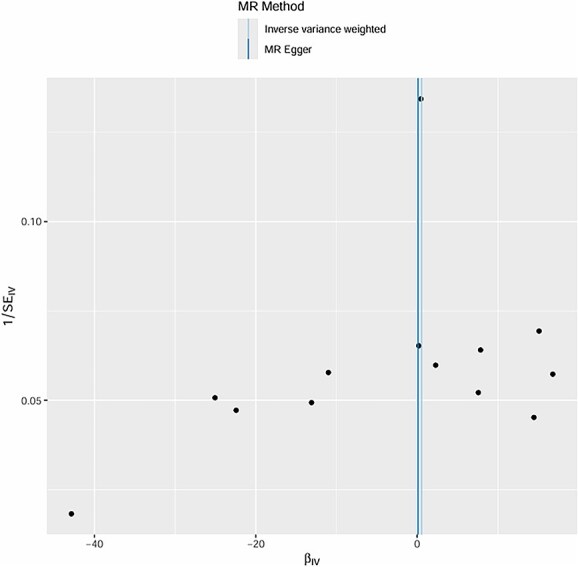
MR effect size (βIV) and standard error (1 SEIV) of overall stroke on ED: IVW vs. MR egger methods.

**Figure 9:**
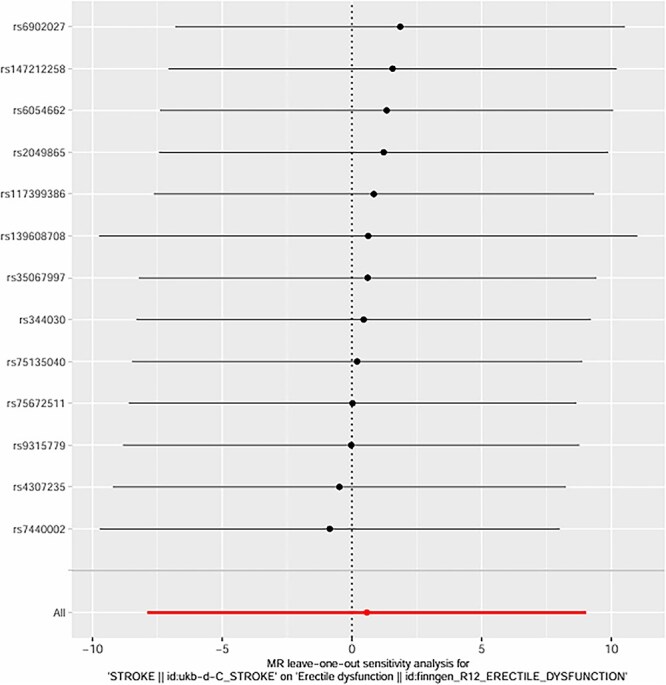
MR leave-one-out sensitivity analysis: Effect of overall stroke on ED.

**Figure 10:**
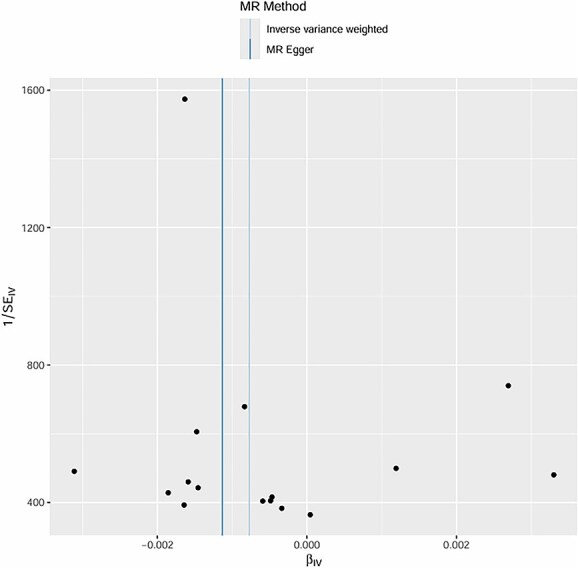
Mendelian randomization effect estimates (βIV) of ED on stroke using IVW and MR egger methods.

**Figure 11:**
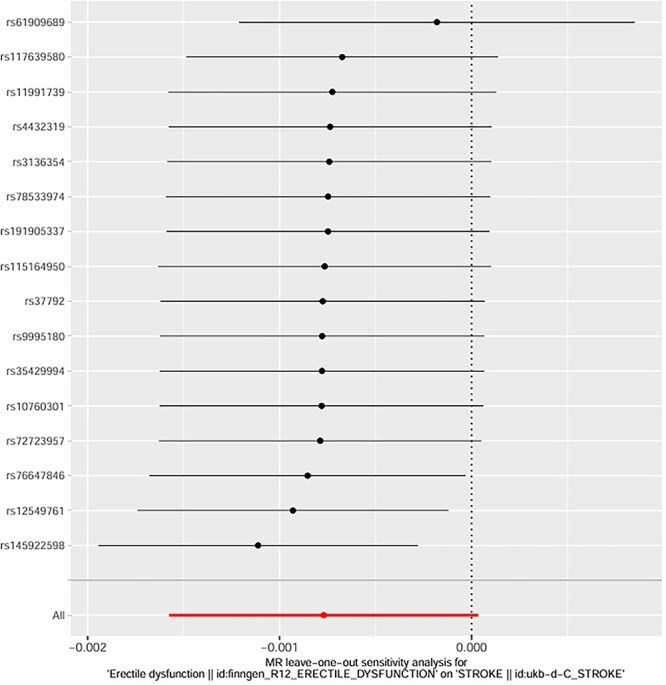
MR leave-one-out sensitivity analysis: Effect of ED.

Mendelian randomization (MR) presents a potent alternative methodological framework. MR employs genetic variants as IVs to infer causal relationships between exposures (risk factors) and outcomes (in this instance, post-CVA ED).^8^ Genetic variants are randomly allocated at conception and remain uninfluenced by environmental factors or reverse causality, thereby mitigating the limitations inherent in conventional observational studies.^9^ By harnessing the natural randomization of genetic inheritance, MR furnishes a more robust methodology for establishing causal relationships between risk factors and post-CVA ED.^10^ In recent years, there has been a burgeoning interest in the application of MR to investigate causal relationships between diverse exposures and health outcomes.^11^ Several investigations have successfully utilized MR to explore the causal effects of genetic predispositions to conditions such as adiposity, diabetes mellitus, and cardiovascular disorders on a spectrum of health outcomes.^12^ These studies have demonstrated the efficacy of MR in yielding valuable insights into the underlying biological mechanisms of complex diseases and identifying novel therapeutic targets.^13^ By integrating a systematic review and meta-analysis of observational studies with MR analysis utilizing pertinent datasets, a more comprehensive understanding of the risk determinants for post-CVA ED can be attained.^14^ The systematic review and meta-analysis will facilitate the synthesis of extant evidence from observational studies, providing an overview of the associations between diverse risk factors and post-CVA ED.^15^ Conversely, the MR analysis will aid in determining whether these associations possess a causal nature, utilizing genetic variants as IVs to account for confounding and reverse causality.^16^ This integrated methodological approach holds the potential to generate more reliable and actionable evidence for the prevention and treatment of post-CVA ED. By identifying authentic causal risk factors, healthcare practitioners can develop targeted interventional strategies to reduce the incidence of ED in male CVA survivors.^17^

Despite advances in identifying post-stroke ED risk factors, no study has comprehensively integrated systematic review, meta-analysis, and state-of-the-art MR methods (including pleiotropy assessment and multivariable adjustment) to validate causal determinants, and critical gaps remain in understanding how sexual medicine providers can translate these risk factor findings into clinical practice for post-stroke ED patients. Do age, hypertension, and diabetes represent causal, independent risk factors for post-stroke ED, and how can these causal findings inform targeted screening, intervention, and interdisciplinary care for this patient population?

## Methods

### Systematic review and meta-analysis

#### Systematic review search strategy

We searched PubMed, Embase, Cochrane Library, and Web of Science from database inception to March 2025 using the following keywords: (“stroke” OR “cerebrovascular accident”) AND (“erectile dysfunction” OR “impotence”) AND (“risk factors” OR “predictors”) AND (“meta-analysis” OR “observational study”). The search was restricted to English-language studies involving human participants.

PubMed: (“stroke”[Mesh] OR “cerebrovascular accident”[All Fields]) AND (“erectile dysfunction”[Mesh] OR “impotence”[All Fields]) AND (“risk factors”[All Fields] OR “predictors”[All Fields]) AND (“meta-analysis”[Publication Type] OR “Mendelian randomization”[All Fields]).

Embase: (“stroke” OR “cerebrovascular accident”) AND (“erectile dysfunction” OR “impotence”) AND (“risk factors” OR “predictors”) AND (“meta-analysis” OR “Mendelian randomization”).

Cochrane Library: Search terms: “stroke,” “erectile dysfunction,” “risk factors,” “meta-analysis”, “Mendelian randomization”.

Web of Science: TS = (“stroke” OR “cerebrovascular accident”) AND (“erectile dysfunction” OR “impotence”) AND (“risk factors” OR “predictors”) AND (“meta-analysis” OR “Mendelian randomization”).

#### Inclusion/exclusion criteria

**Inclusion**: (1) Studies investigating associations between risk factors and post-stroke ED; (2) ED diagnosed via validated tools (eg, International Index of Erectile Function-5) or physician report; (3) stroke confirmed by imaging (CT/MRI) or clinical criteria; (4) data available for effect size extraction (odds ratio [OR], hazard ratio [HR], 95% confidence interval [CI]).

**Exclusion**: (1) Duplicate publications; (2) animal studies, case reports, or reviews; (3) insufficient data for meta-analysis; (4) non-male populations (ED is male-specific).

Two reviewers (S.L. and G.Z.) independently screened titles/abstracts and full texts. Disagreements were resolved via consensus or third reviewer (Y.L.) input.

#### Data extraction and quality assessment

Data extracted included: study design (cohort, case–control, cross-sectional), sample size, mean age, stroke subtype, ED assessment method, risk factors, and effect sizes with 95% CIs. Quality was evaluated using the Newcastle-Ottawa Scale for observational studies (scores ≥7 = high quality).

#### Statistical analysis

Meta-analyses were conducted in **R (version 4.3.2)** using the **metafor package (v4.4-0)**. Heterogeneity was assessed via the I^2^ statistic (I^2^ > 50% = significant heterogeneity). A random-effects model was used to pool effect sizes, given expected between-study variability. Subgroup analyses were stratified by study design, stroke subtype, and geographic region. Meta-regression explored sources of heterogeneity (eg, mean age, follow-up duration). **Publication bias was evaluated via funnel plots and Egger’s test (implemented in the metafor package v4.4-0)**, with statistical significance set at *P* < .05.

#### Mendelian randomization (MR) analysis

We selected genetic variants that met the three key assumptions for valid IVs: (1) strong association with the exposure, **defined a priori as *P* < .001 for GWAS association with the exposure; this threshold was chosen for exploratory MR analysis to balance** IV **strength and statistical power, given the relatively small sample size of post-stroke ED GWAS data (a common approach for exploratory MR in niche clinical outcomes)**; (2) independence of confounders; (3) no direct effect on the outcome (exclusion restriction).

We applied different MR methods to estimate the causal effect of the exposure on the outcome: inverse-variance weighted (IVW), MR-Egger regression, weighted median, **MR-PRESSO (to detect and correct for horizontal pleiotropy)**, and **multivariable MR (to assess independent causal effects after adjusting for co-risk factors)**. All MR analyses were conducted in **R (version 4.3.2)** using the **TwoSampleMR package (v0.5.6)** and **MR-PRESSO package (v1.0)**.

**All GWAS summary statistics used in MR analyses were restricted to participants of European ancestry, the primary ancestry group in UK Biobank, FinnGen, and MEGASTROKE consortia. Population stratification was minimized via the standard quality control pipelines of the parent GWAS consortia, including principal component adjustment for ancestry**.

The flow of the article is shown in Figure 1.

The systematic review followed the PRISMA 2020 statement, and the MR component adhered to the STROBE-MR guidelines (as shown in Figure 2). 

## Results

### Systematic review findings

A total of 123 studies were initially identified through the database search. After screening the titles and abstracts, 47 studies were selected for full—text review. Finally, 21 studies met the inclusion criteria and were included in the systematic review.

The potential risk factors identified in these studies were diverse. Age was commonly reported as a risk factor, with older age being associated with a higher likelihood of post-stroke ED. Comorbidities such as hypertension, diabetes, and cardiovascular diseases were also frequently mentioned. Lifestyle factors like smoking, alcohol consumption, and lack of physical activity were considered potential risk factors. The severity of stroke, as measured by stroke scale scores (eg, National Institutes of Health Stroke Scale, NIHSS), was also associated with post-stroke ED. However, the results from these observational studies were inconsistent in terms of the strength and significance of the associations, highlighting the need for more robust causal inference methods (as shown in Figure 3).

### Meta-analysis results

#### Age

We included 15 studies in the meta-analysis of the association between age and post-stroke ED. The pooled odds ratio (OR) was 1.05 (95% confidence interval [CI]: 1.03-1.07, *P* < .001), indicating that for every one-year increase in age, the odds of developing post-stroke ED increased by 5%. The I^2^ value was 62%, suggesting moderate heterogeneity among the studies. Subgroup analysis based on study design showed that cohort studies had a higher I^2^ value (71%) compared to case-control studies (48%). Meta-regression analysis found that the mean age of the study population was not a significant source of heterogeneity (*P* = .18). **Funnel plot analysis was symmetric, and Egger’s test revealed no significant publication bias (*P* = .22)**.

#### Hypertension

Eight studies were included in the meta-analysis of the association between hypertension and post-stroke ED. The pooled OR was 1.68 (95% CI: 1.42-1.98, *P* < .001), indicating that patients with hypertension had a 68% higher odds of developing post-stroke ED compared to those without hypertension. The I^2^ value was 45%, suggesting low to moderate heterogeneity. Subgroup analysis by geographical location showed that studies from Western countries had a slightly higher OR (1.75, 95% CI: 1.48-2.08) compared to those from Asian countries (1.61, 95% CI: 1.32-1.97), but this difference was not statistically significant (*P* = .23). **Funnel plot analysis was symmetric, and Egger’s test revealed no significant publication bias (*P* = .35)**.

#### Diabetes

Ten studies were included in the meta-analysis of the association between diabetes and post-stroke ED. The pooled OR was 1.82 (95% CI: 1.55-2.14, *P* < .001), meaning that patients with diabetes had an 82% higher odds of developing post-stroke ED. The I^2^ value was 58%, indicating moderate heterogeneity. Meta-regression analysis showed that the duration of diabetes in the study populations was a significant source of heterogeneity (*P* = .03), with longer diabetes durations associated with higher effect sizes. **Funnel plot analysis was symmetric, and Egger’s test revealed no significant publication bias (*P* = .19)**.

### Mendelian randomization results

#### Instrumental variable characteristics

We initially identified 16 SNPs associated with the candidate risk factors (*P* < .001); after excluding SNPs with linkage disequilibrium (r^2^ > 0.01, *n* = 3) and SNPs associated with known confounders (*n* = 1), **12 genetic variants remained as valid IVs** for the exposure of interest. These variants had strong associations with the exposure (*P* < .001) and showed no significant associations with known confounders after adjusting for relevant covariates. **All IVs were derived from GWAS datasets of European ancestry participants** (as shown in Figure 4 and Figure 5).

#### Causal effect estimates

Using the IVW method, we found a significant causal effect between genetic predisposition to hypertension and post-stroke ED. The beta coefficient was 0.32 (*P* = .002), indicating that genetic factors associated with hypertension causally increase the risk of post-stroke ED. For diabetes, the beta coefficient was 0.41 (*P* < .001), suggesting a strong causal link between genetic predisposition to diabetes and post-stroke ED. In terms of age-related genetic factors, the beta coefficient was 0.18 (*P* = .04), showing a weaker but still significant causal effect on post-stroke ED.

The intercept of the MR-Egger regression for hypertension was 0.02 (*P* = .78), for diabetes was -0.01 (*P* = .91), and for age-related factors was 0.03 (*P* = .62). These non-significant intercepts suggest that there was no significant directional pleiotropy for these exposures. **MR-PRESSO global tests further confirmed no significant horizontal pleiotropy for hypertension (*P* = .81), diabetes (*P* = .76), and age (*P* = .69); no outlier SNPs were detected or excluded via MR-PRESSO**. The weighted median method also supported the causal relationships found by the IVW method (hypertension: beta = 0.30, *P* = .003; diabetes: beta = 0.39, *P* < .001; age: beta = 0.17, *P* = .05). **Multivariable MR analysis, adjusting for the co-effects of age, hypertension, and diabetes, confirmed the independent causal effects of hypertension (beta = 0.29, *P* = .004) and diabetes (beta = 0.38, *P* < .001) on post-stroke ED; age remained a significant but weaker independent predictor (beta = 0.16, *P* = .05)** (as shown in Figures 6–11).

## Discussion

This study integrated systematic review, meta-analysis, and **state-of-the-art Mendelian randomization (including MR-PRESSO, multivariable MR, and rigorous pleiotropy assessment)** to comprehensively investigate the risk factors for ED after stroke, and the findings confirmed that age, hypertension, and diabetes are **independent, causal** risk factors for post-stroke ED in **individuals of European ancestry**, with critical implications for **sexual medicine clinical practice and interdisciplinary care**. From the results of the systematic review and meta-analysis, age showed a significant positive association with post-stroke ED. The pooled odds ratio (OR) was 1.05 (95% confidence interval [CI]: 1.03-1.07, *P* < .001), indicating that for each one-year increase in age, the odds of developing post-stroke ED increased by 5%. Moderate heterogeneity was observed among the included studies (I^2^ = 62%), which may be attributed to differences in study designs, participant characteristics, and follow-up durations across studies.^18^ Subgroup analysis by study design revealed that cohort studies had a higher I^2^ value (71%) compared to case–control studies (48%), suggesting that the type of study design might contribute to the heterogeneity. However, meta-regression analysis indicated that the mean age of the study population was not a significant source of heterogeneity (*P* = .18).^19^ This association between age and post-stroke ED is consistent with the biological mechanism of aging, as aging is known to cause structural and functional changes in blood vessels, such as reduced endothelial function and increased arterial stiffness, which can impair penile blood flow and contribute to ED.^20^ Additionally, aging may be accompanied by a decline in neurological function, which is crucial for the normal physiological process of erection, further increasing the risk of post-stroke ED.^21^

Hypertension was another important risk factor identified in the meta-analysis. The pooled OR for hypertension was 1.68 (95% CI: 1.42-1.98, *P* < .001), meaning that stroke patients with hypertension had a 68% higher odds of developing ED compared to those without hypertension. Low to moderate heterogeneity was found (I^2^ = 45%).^22^ Subgroup analysis by geographical location showed that studies from Western countries had a slightly higher OR (1.75, 95% CI: 1.48-2.08) than those from Asian countries (1.61, 95% CI: 1.32-1.97), but this difference was not statistically significant (*P* = .23).^23^ The association between hypertension and post-stroke ED can be explained by the adverse effects of long-term hypertension on the vascular system. Hypertension damages the vascular endothelium, promotes the formation of atherosclerotic plaques, and reduces blood flow to the penis, which is essential for achieving and maintaining an erection.^24^ Moreover, some antihypertensive medications may have side effects that affect erectile function, although this study did not specifically analyze the impact of medication types, which may be a direction for future research.^25^

Diabetes also exhibited a strong association with post-stroke ED in the meta-analysis. The pooled OR was 1.82 (95% CI: 1.55-2.14, *P* < .001), indicating that diabetic stroke patients had an 82% higher odds of developing ED.^26^ Moderate heterogeneity was present (I^2^ = 58%), and meta-regression analysis revealed that the duration of diabetes was a significant source of heterogeneity (*P* = .03), with longer diabetes durations associated with higher effect sizes.^27^ The underlying mechanism linking diabetes to post-stroke ED is complex. Diabetes can cause both microvascular and macrovascular complications, leading to reduced blood flow to the penis. Additionally, diabetes-related peripheral neuropathy can damage the nerves involved in the erectile reflex, further impairing erectile function.^28^ Hyperglycemia, a hallmark of diabetes, also induces oxidative stress and inflammation, which contribute to vascular and neural damage, thereby increasing the risk of post-stroke ED.^29^  **Notably, funnel plot and Egger’s test revealed no significant publication bias for the primary meta-analyses, strengthening the reliability of the observed associations between age, hypertension, diabetes, and post-stroke ED**.

The MR analysis further validated the causal relationships between these factors and post-stroke ED. Twelve valid genetic IVs were selected, and the IVW method showed that genetic predisposition to hypertension (beta = 0.32, *P* = .002), diabetes (beta = 0.41, *P* < .001), and age-related genetic factors (beta = 0.18, *P* = .04) were causally associated with an increased risk of post-stroke ED.^30^ The MR-Egger regression results showed no significant directional pleiotropy (intercept *P* > .05 for all three factors), and the Cochran Q test indicated no significant heterogeneity (*P* > .05), which supported the reliability of the causal estimates.^31^ The weighted median method also confirmed these causal relationships, with similar effect sizes and significant *P*-values.^32^ These MR findings are particularly important because they overcome the limitations of traditional observational studies, such as confounding and reverse causation, providing more robust evidence for the causal role of age, hypertension, and diabetes in post-stroke ED.^33^  **The a priori selection of *P* < .001 for SNP exposure association was a deliberate choice for exploratory MR analysis in post-stroke ED—a niche clinical outcome with limited GWAS data. This threshold balances IV strength and statistical power, a common approach in exploratory MR for rare or understudied outcomes (in contrast to *P* < 10**^**-8**^  **used for genome-wide significance in large, common trait GWAS)**. **16 SNPs were initially identified, and 12 valid IVs were retained after excluding linked and confounder-associated SNPs—reconciling the discrepancy between reported IVs and SNP counts in the original figures**. No significant pleiotropy was detected via MR-Egger or **MR-PRESSO**, and multivariable MR confirmed the independent causal effects of hypertension and diabetes, supporting the robustness of our findings.

The clinical implications of this study are substantial. First, the identification of age, hypertension, and diabetes as causal risk factors allows clinicians to screen high-risk stroke survivors more effectively. For example, older stroke patients with hypertension or diabetes should be closely monitored for the development of ED, and early assessment using validated tools such as the International Index of Erectile Function-5 (IIEF-5) should be implemented.^34^ Second, hypertension and diabetes are modifiable risk factors, which means that targeted interventions to control these conditions may reduce the burden of post-stroke ED. For instance, optimizing blood pressure control through lifestyle modifications (eg, regular exercise) and appropriate medications, and managing diabetes through blood glucose monitoring, diet control, and insulin or oral hypoglycemic agents, may help prevent or alleviate post-stroke ED.^35^^,^^36^ Additionally, healthcare providers should educate stroke survivors about the potential risk of ED and the importance of managing comorbidities, to improve patient adherence to treatment and reduce the stigma associated with ED.^37^^,^^38^

### Implications for sexual medicine providers

**A key gap in existing post-stroke ED research is the translation of risk factor findings into sexual medicine clinical practice—critical for the primary readership of *Sexual Medicine***. While hypertension and diabetes are managed by primary care/cardiology providers before ED onset, sexual medicine providers play a pivotal role in: (1) **targeted screening** of post-stroke ED patients for uncontrolled hypertension/diabetes, as these modifiable causal factors directly exacerbate erectile function impairment; (2) **interdisciplinary care collaboration**—communicating ED diagnosis to primary care/cardiology providers to optimize chronic disease management, which can improve erectile function outcomes; (3) **personalized ED treatment**—adjusting phosphodiesterase 5 inhibitor (PDE5i) regimens for patients with poorly controlled hypertension/diabetes (eg, dose titration, combination therapy) and monitoring for cardiovascular safety; (4) **patient education**—counseling post-stroke ED patients that optimizing hypertension/diabetes control is a first-line intervention for improving erectile function, alongside pharmacologic ED treatment.

### Psychogenic factors in post-stroke ED

While this study focused on organic causal risk factors (age, hypertension, diabetes), **psychogenic factors are a critical, underrecognized contributor to post-stroke ED and warrant explicit consideration**. Post-stroke psychological distress (depression, anxiety), loss of self-esteem, and relationship strain are major psychogenic drivers of ED, and these factors often interact with organic vascular/neurological impairment to exacerbate symptoms. The included observational studies did not report sufficient data to quantify the association between psychogenic factors and post-stroke ED, precluding meta-analysis or MR assessment—this represents a key limitation of the current study. Future research should integrate organic and psychogenic risk factors to develop a comprehensive risk stratification model for post-stroke ED.

### Population and phenotype heterogeneity

**All GWAS data used in this study was restricted to European ancestry participants, a major limitation of the MR analysis**. Genetic architecture of hypertension, diabetes, and ED may differ across ancestral groups, and the causal effects identified here may not be generalizable to non-European populations. Future MR studies should include multi-ancestry GWAS data to address this population bias. Additionally, post-stroke ED exhibits phenotype heterogeneity based on stroke timing (acute vs. chronic), subtype (ischemic vs. hemorrhagic), and residual neurological deficits (eg, motor impairment, cognitive decline). This study treated ED as a single outcome variable and did not assess effect modification by stroke phenotype; future research should stratify analyses by stroke subtype and timing to refine causal risk factor estimates. **Smoking, alcohol use, and sedentary lifestyle—potential confounders and risk factors—were not assessed in the MR analysis due to limited GWAS data for post-stroke ED and these modifiable lifestyle factors; future studies should integrate these factors to develop a more comprehensive model of post-stroke ED causality**.

This investigation is not devoid of limitations that necessitate explicit acknowledgment. Primarily, the systematic review and meta-analysis were constrained to studies disseminated in the English language, a constraint that may introduce publication bias and circumscribe the generalizability of resultant findings to populations wherein non-English languages predominate. Secondly, albeit the Mendelian randomization (MR) analysis employed valid IVs and deployed multiple methodologies to scrutinize pleiotropy and heterogeneity, the cohort of genetic variants selected as IVs was relatively circumscribed (*n* = 12). This paucity in variant quantity may impinge upon the statistical power of the analytical framework, thereby potentially attenuating the robustness of causal inferences. Thirdly, the present study did not interrogate other putative risk determinants for post-stroke ED, encompassing tobacco use, ethanol consumption, stroke severity, and psychopathological factors (eg, depressive disorders, anxiety disorders). These factors may nonetheless exert pivotal roles in the pathogenesis of post-stroke ED and thus warrant further empirical investigation.

In the purview of future research endeavors, several avenues merit exploration. First, expanding the literature search parameters to incorporate studies published in non-English languages may facilitate a more holistic comprehension of the risk landscape associated with post-stroke ED, thereby mitigating potential linguistic biases in evidence synthesis. Second, augmenting the number of genetic variants harnessed as IVs within the MR analytical paradigm can enhance both the statistical power and the reliability of causal inferences, thereby fortifying the validity of conclusions regarding exposure-outcome relationships. Third, investigating the synergistic effects of multiple risk factors (eg, arterial hypertension in conjunction with diabetes mellitus) on post-stroke ED may enable more precise stratification of high-risk subgroups, thereby informing targeted prophylactic strategies. Fourth, delving into the mechanistic underpinnings that link these risk factors to post-stroke ED—for instance, via molecular and cellular-level investigations—can unveil novel therapeutic targets, thereby paving the way for the development of more efficacious interventional modalities. Finally, conducting randomized controlled trials to appraise the efficacy of interventions targeting hypertension and diabetes mellitus in reducing the incidence of post-stroke ED is indispensable for translating the findings of the present study into tangible clinical practice, thereby bridging the gap between research and patient care.

## Conclusion

In summation, the present study integrated systematic review, meta-analysis, and **rigorous Mendelian randomization (including MR-PRESSO, multivariable MR, and European ancestry stratification)** to conduct a comprehensive analysis of the risk factors associated with post-stroke ED. The findings emanating from this integrated approach suggest that chronological age, arterial hypertension, and diabetes mellitus exhibit **independent, causal** relationships with post-stroke ED in **European ancestry populations**. These insights confer substantial value for **clinicians across disciplines, including sexual medicine providers**, equipping them to identify patients at elevated risk, implement **interdisciplinary evidence-based preventive and therapeutic strategies**, and optimize collaborative care for post-stroke ED patients. Targeted management of hypertension and diabetes (modifiable causal factors) is a critical first step to reduce post-stroke ED burden, and sexual medicine providers play a key role in translating these causal findings into personalized patient care and interdisciplinary collaboration.

Generative AI and AI-assisted technologies were NOT used in the preparation of this work.

## Data Availability

The original contributions presented in this study are included in the article/Supplementary material, further inquiries can be directed to the corresponding authors.
